# Enhancing Thermal and Mechanical Properties of Ramie Fiber via Impregnation by Lignin-Based Polyurethane Resin

**DOI:** 10.3390/ma14226850

**Published:** 2021-11-13

**Authors:** Sucia Okta Handika, Muhammad Adly Rahandi Lubis, Rita Kartika Sari, Raden Permana Budi Laksana, Petar Antov, Viktor Savov, Milada Gajtanska, Apri Heri Iswanto

**Affiliations:** 1 Department of Forest Products, Faculty of Forestry and Environment, IPB University, Bogor 16680, Indonesia; sucia_okta@apps.ipb.ac.id; 2 Research Center for Biomaterials, National Research and Innovation Agency, Cibinong 16911, Indonesia; raden.budi.biomaterial@gmail.com; 3 Faculty of Forest Industry, University of Forestry, 1797 Sofia, Bulgaria; p.antov@ltu.bg (P.A.); victor_savov@ltu.bg (V.S.); 4 Faculty of Wood Sciences and Technology, Technical University in Zvolen, 96001 Zvolen, Slovakia; 5 Department of Forest Product, Faculty of Forestry, Universitas Sumatera Utara, Medan 20155, Indonesia; apri@usu.ac.id

**Keywords:** fractionated lignin, bio-polyurethane resin, ramie fiber, impregnation, thermal stability, mechanical properties

## Abstract

In this study, lignin isolated and fractionated from black liquor was used as a pre-polymer to prepare bio-polyurethane (Bio-PU) resin, and the resin was impregnated into ramie fiber (*Boehmeria nivea* (L.) Gaudich) to improve its thermal and mechanical properties. The isolated lignin was fractionated by one-step fractionation using two different solvents, i.e., methanol (MeOH) and acetone (Ac). Each fractionated lignin was dissolved in NaOH and then reacted with a polymeric 4,4-methane diphenyl diisocyanate (pMDI) polymer at an NCO/OH mole ratio of 0.3. The resulting Bio-PU was then used in the impregnation of ramie fiber. The characterization of lignin, Bio-PU, and ramie fiber was carried out using several techniques, i.e., Fourier-transform infrared spectroscopy (FTIR), differential scanning calorimetry (DSC), thermogravimetric analysis (TGA), pyrolysis-gas-chromatography-mass-spectroscopy (Py-GCMS), Micro Confocal Raman spectroscopy, and an evaluation of fiber mechanical properties (modulus of elasticity and tensile strength). Impregnation of Bio-PU into ramie fiber resulted in weight gain ranging from 6% to 15%, and the values increased when extending the impregnation time. The reaction between the NCO group on Bio-PU and the OH group on ramie fiber forms a C=O group of urethane as confirmed by FTIR and Micro Confocal Raman spectroscopies at a wavenumber of 1600 cm^−1^. Based on the TGA analysis, ramie fiber with lignin-based Bio-PU had better thermal properties than ramie fiber before impregnation with a greater weight residue of 21.7%. The mechanical properties of ramie fiber also increased after impregnation with lignin-based Bio-PU, resulting in a modulus of elasticity of 31 GPa for ramie-L-isolated and a tensile strength of 577 MPa for ramie-L-Ac. The enhanced thermal and mechanical properties of impregnated ramie fiber with lignin-based Bio-PU resins could increase the added value of ramie fiber and enhance its more comprehensive industrial application as a functional material.

## 1. Introduction

Polyurethane (PU) resin is a polymer that contains urethane linkages (R−N−H−C=O−R) in the main polymer chain. PU can be functionalized with reactive groups such as isocyanate, amine, epoxy, acrylate, or carboxylic acid groups [1]. Modified PU resins are widely used in various applications, such as coatings, foams, medicinal products, thermal insulation, and adhesives [2,3,4,5]. Due to its superior properties compared to other polymers, such as high mechanical strength, flexibility at low temperatures, and the ability to be formed as a rigid, semi-rigid, or flexible foam with various densities, PU has become one of the most important type of resins used in various value-added applications, with an estimated global market volume of approximately 24 million tons in 2020 [6,7]. The synthesis of PU is carried out through a condensation reaction between isocyanates and polyols, where the main ingredients of the polymer to produce polyurethane are derived from refining crude oil and coal [8]. Hence, the main environmental problem in the production process is related to the formation of toxic substances such as polyisocyanate and its phosgene intermediate. The increased industrial PU demands and growing environmental concerns have posed new requirements related to the utilization of raw materials from renewable, bio-based sources to reduce declining fossil reserves. The environmental approaches in the development of sustainable PUs have resulted in the replacement of petrochemical polyols with bio-polyols, such as vegetable oil, oil waste, or lignocellulosic biomass [9]. Major techniques for converting vegetable oils to polyols are hydroformylation, transesterification/amidation, epoxidation/oxirane-ring opening, and ozonolysis [10,11,12,13]. Lignocellulosic biomass can be converted to liquid polyols through liquefaction processes or oxypropylation for polyurethane applications [14]. Bio-polyurethanes (Bio-PU) can also be obtained by using polyols from renewable materials such as lignin and tannin for various applications [15,16].

Lignin is the second most abundant polymer source in nature after cellulose, accounting for around 15–30% in wood biomass (bark typically contains large amounts of lignin ca. 40%) and 10–20% in grass biomass [17,18,19]. Nowadays, lignin is mainly produced as a waste or by-product of the pulp and paper industry with an estimated annual volume of about 50–70 million tons of which less than 2% is converted into value-added applications [20,21]. Lignin has a heterogeneous, amorphous structure, and consists of three-dimensional non-uniform polymeric tissue, constructed with repeating units [9]. Lignin is a complex aromatic polymer of three types of phenylpropane units (monolignols), namely p-hydroxyphenyl, syringyl, and guaiacyl. These units are randomly linked by ether and carbon bonds, forming a three-dimensional structural network with several reactive groups in structures, such as phenolic/aliphatic hydroxyl, methoxyl, carbonyl, and carboxyl [22]. The hydroxyl group and free position in the aromatic rings are the principal functions of lignin.

Lignin has emerged as a good alternative to polyol in resins, foams, and adhesives The NCO content of lignin Bio-PU decreases compared to the unmodified PU batch, which indicates that the incorporation of lignin in Bio-PUs leads to efficient crosslinking and the presence of less monomer in the final system. The incorporation of lignin results in an increased molecular weight of the lignin Bio-PU, which leads to the successful incorporation of lignin into the backbone of the polyurethane chain. Thus, the overall molecular weight of the adhesive is also increased [22]. Lignin-based, cross-linked PUs prepared from low- and medium-molecular-weight lignin fractions are characterized by high tensile strength and a high modulus of elasticity. However, their brittleness increases with the increased lignin content. The molecular weight of lignin can be altered by crosslinking with various solvents, which can tune the mechanical properties of the lignin-based PUs. Methanol fractionation of lignin increases the molecular weight (by removing the low-molecular-weight fraction), polydispersity, char yield, and glass transition temperature, and acetone fractionation improves the role of lignin as an antioxidant. Acetone-soluble lignin fractionation-based PU exhibits improved thermal stability due to crosslinking the lignin macromonomers [23].

The use of lignin as a Bio-PU pre-polymer follows two approaches: Lignin used directly without chemical modification or combined with other polymers, and using lignin after chemical modifications, such as esterification and etherification reactions to ensure hydroxyl groups react more easily [24]. Isolated lignin has aliphatic and hydroxyl phenolic groups, which provide excellent bonding to isocyanates. Most of the phenolic hydroxyls are linked to the adjacent phenylpropane units. In addition, chemical modifications such as methylolation and phenolation increase its chemical reactivity to formaldehyde and thus lignin can also act as a formaldehyde scavenger in conventional thermosetting formaldehyde-based adhesives and has unique characteristics with comprehensive strength and thermal stability because of the non-crystalline network structure [25,26,27,28].

Ramie (*Boehmeria nivea* (L.) Gaudich) is an abundant natural fiber that is easy to obtain, renewable, and environmentally sustainable as a resource material. Ramie fiber exhibits satisfactory strength and stiffness properties, low cost, and relatively high annual production [29]. Ramie fiber also has good decay resistance and the ramie-based composite products have twice the strength of regular wood fiber [30]. Ramie fiber has a tensile strength of around 95 MPa, which is higher than cotton and hemp [31]. Ramie fiber contains cellulose, hemicellulose, lignin, ash, and wax in different amounts, which cause differences in physical, mechanical, and thermal properties between ramie fiber and other cellulosic fibers. To obtain optimal composite characteristics, it is necessary to select the physical, mechanical, and thermal properties of the raw materials used [32]. Ramie fiber has been widely used in automotive, interior design, and furniture applications [33]. However, the main disadvantages of ramie fiber are its low thermal stability and poor flame retardancy, which limit the use of ramie as a raw material for functional textiles. Chemical treatments such as alkali, acetylation, and bleaching [34], as well as biological treatments have been performed to overcome those drawbacks [35]. In addition, the mechanical properties of ramie fiber often decrease due to the removal of cellulose, hemicellulose, and lignin after modifications.

One way to enhance both the thermal stability and mechanical properties of ramie fiber is by using the impregnation method. Impregnation is an important method in composite manufacturing. Different types of natural fibers have been impregnated with polypropylene, polylactic acid, and aluminum hydroxide [36,37]. The use of lignin derived from black liquor as polyols in the synthesis of bio-PU represents a sustainable approach to reduce waste from the kraft pulping process. The resulting bio-PU was used as an impregnation material aimed at improving the fiber thermal and mechanical characteristics. The impregnation of natural fibers can be simplified as a flow of viscous chemicals into porous materials. However, to the best of the authors’ knowledge, no studies have reported the impregnation of ramie fiber to enhance its thermal and mechanical properties.

Therefore, the aim of this research work was to develop bio-PU resin derived from lignin for the impregnation of ramie fiber and investigate the properties of Bio-PU resin and the performance of impregnated ramie fiber, particularly its thermal stability and mechanical properties. The analyses used included functional group analysis using Fourier-transform infrared spectroscopy (FTIR), thermal analysis using Differential Scanning Calorimetry (DSC), thermal stability analysis using Thermogravimetric (TGA), and Pyrolysis-Gas-Chromatography-Mass-Spectroscopy (Py-GCMS) for determining lignin components, and the evaluation of tensile strength and the modulus of elasticity of ramie fiber using a universal testing machine (UTM).

## 2. Materials and Methods

### 2.1. Materials

**Figure 1:**
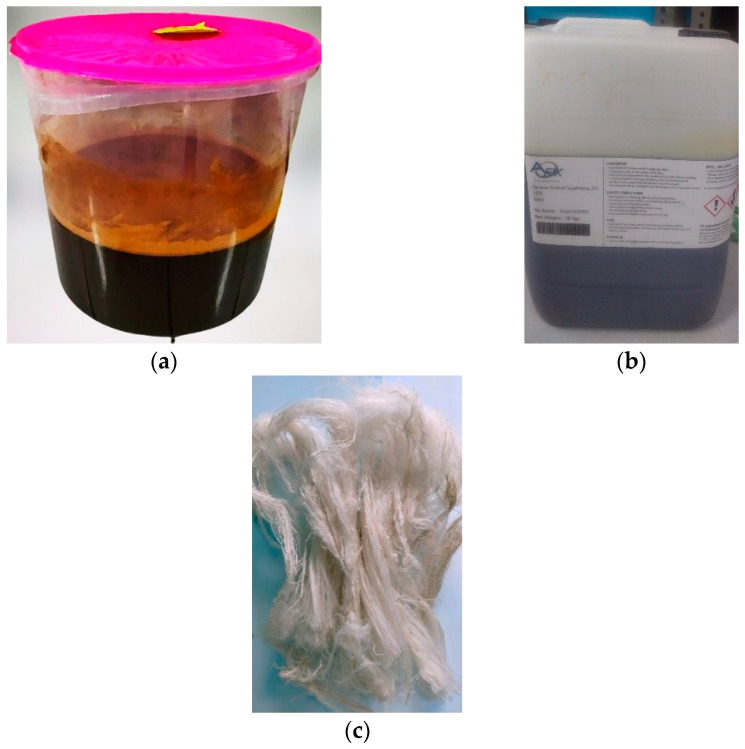
The materials used (**a**) black liquor; (**b**) pMDI; (**c**) ramie fibers.

Black liquor obtained from the kraft pulping process of *Acacia mangium* was provided by Tanjung Enim Lestari Pulp and Paper Company, Muara Enim, Indonesia (Figure 1). Standardized kraft lignin (guaiacyl lignin) from Sigma Aldrich (CAS No. 8068-05-1, Burlington, MA, USA) was used as a standard. Ramie fiber (*Boehmeria nivea* (L.) Gaudich) was obtained from Rabersa Company, Wonosobo, Indonesia. Hydrochloric acid (HCl 37%, analytical grade, Merck, Darmstadt, Germany), sulfuric acid (H_2_SO_4_ 95–97%, analytical grade, Merck, Darmstadt, Germany), methanol (analytical grade, Merck, Darmstadt, Germany), acetone (analytical grade, Merck, Darmstadt, Germany), dioxane (1,4-dioxane, analytical grade, Merck, Darmstadt, Germany), and distilled water were used for isolation and fractionation of lignin. Polymeric 4,4-methane diphenyl diisocyanate (pMDI, ±31% NCO content), purchased from the company Anugerah Raya Kencana (ARK, Tangerang, Indonesia), and sodium hydroxide (NaOH 20%) were used to prepare the Bio-PU resin.

### 2.2. Isolation and Fractionation of Lignin

Lignin was isolated via the acid precipitation method [38]. Approximately 200 g of black liquor and 2000 mL of distilled water were stuffed into a plastic jar. HCl 1 M was added while stirring to adjust the pH of black liquor from around 12 to 2. The solution was kept for 24 h at room temperature (25 °C) to separate the filtrate and residue. Further, the decantation process was carried out three times, and then the remaining sludge was refrigerated for 24 h. The precipitate was filtered with a Buchner funnel and kept in an oven for 24 h at 45 °C. Then the precipitate was powdered using a mortar and filtered with a size 60 mesh.

Lignin fractionation was carried out via a single-step fractionation using two different solvents, namely methanol (MeOH) and acetone (Ac) with some modifications [39]. The lignin sample was weighed and 20 g was mixed with 300 mL of solvent at a ratio of 1:15. The solution then was stirred with a magnetic stirrer for 24 h with a stirring speed of 200 rpm. The soluble and insoluble fractions were separated using a Buchner funnel and filter paper. After that, the dissolved fraction was concentrated with a rotary evaporator (Rotavapor^®^ R-300, Buchi, Flawil, Switzerland). Furthermore, the lignin samples were dried for 24 h at 45 °C.

### 2.3. Lignin Characterization

Basic properties of lignin such as yield, moisture content, ash content, purity, and total phenolic hydroxyl groups were characterized using various analytical methods. The yield of lignin was calculated by dividing the weight of isolated lignin and black liquor. The moisture content of lignin was determined by drying lignin samples in an oven at 105 °C for 24 h.

The ash content of lignin was determined by heating the sample in a furnace for 6 h at a temperature of 525 ± 25 °C. Ash content of lignin was calculated according to the following equation [40].

(1) $$\mathrm{Ash}\;\mathrm{content}\;\left(\%\right)=\frac{\left(C-A\right)}{B}\times 100\%$$

Description:

*A* = weight of empty porcelain cup (g).

*B* = weight of lignin sample (g).

*C* = weight of oven-dried sample and porcelain cup (g).

The purity of isolated lignin was measured according to the standard procedure [41,42]. Approximately 0.3 g of lignin was added to a vial bottle. After that, 3 mL of H_2_SO_4_ 72% was added and mixed with a magnetic stirrer at 150 rpm for 2 h. A blank solution was prepared by adding 3 mL of H_2_SO_4_ 72% and 84 mL of distilled water. Furthermore, the sample was heated by autoclave for 1 h at a temperature of 121 °C. The solution was filtered using an IG3 filter glass (CTE33, IWAKI, Tohoku, Japan). The residue (acid-insoluble lignin) was put in the oven at 105 °C for 24 h. The filtrate was diluted in a test tube with a blank solution of 13 times dilution. The solution was stirred with a vortex, and its absorbance was measured using a UV-Vis spectrophotometer (UV-1800, Shimadzu, Kyoto, Japan) at a wavelength of 240 nm. Acid soluble lignin (ASL) and acid-insoluble lignin (AIL) were calculated using the following equations [41,42].

(2) $$\mathrm{ASL}\;\left(\%\right)=\frac{UVabs\times volume\;filtrate\times dilution}{\varepsilon \times A\times cuvette\;length}\times 100\%$$

(3) AIL (%)=AIR(%)−Ash content (%)

(4) $$\mathrm{Acid}\;\mathrm{Insoluble}\;\mathrm{Residue}\;\left(\mathrm{AIR}\right)\;\left(\%\right)=\frac{C-B}{A}\times 100\%$$

Description:

*ε* = absorptivity constant of biomass at specific wavelength (L/g·cm).

*A* = weight of sample without moisture content (g).

*B* = dry weight of IG3 filter glass (g).

*C* = dry weight of IG3 filter glass and AIL (g).

The total phenolic hydroxyl groups of lignin were determined according to the published methods [43]. The lignin sample was prepared by dissolving 20 mg of lignin in 10 mL of dioxane and 10 mL of NaOH 0.2 M. The solution was filtered using 0.45 μm nylon to remove insoluble particles. Further, 4 mL of the initial solution was diluted by adding 50 mL of solvents. Three different solvents were used: NaOH 0.2 M, pH 6 buffer, and pH 12 buffer. Dilution was carried out until the final concentration of the solution was 0.08 g/L. UV measurements were carried out using a UV-Vis spectrophotometer in the 200–600 nm range using a buffer solution of pH 6 as a reference. The maximum absorption occurred at the absorbance of 300 nm and 350 nm, which were used for calculations. The total phenolic hydroxyl group was calculated using the following equation [43]:

Total OH (mmol g^−1^) = (0.425 × *A*_300nm_ (NaOH) + 0.182 × *A*_350nm_ (NaOH)) × *a* (5)

Description:

*A* = absorbance.

*a* = correction term (L/g·cm) = 1/(*c* × *l*) × 10/17.

*c* = concentration of lignin solution (g/L).

*l* = path length (cm).

### 2.4. Preparation of Bio-Polyurethane Resin

**Figure 2:**
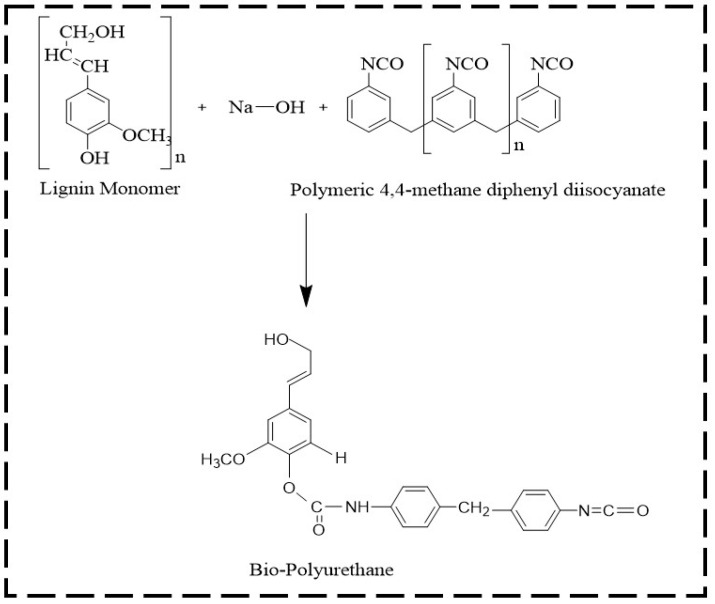
The possible reaction scheme of lignin-based Bio-PU resin.

Bio-polyurethane (Bio-PU) was prepared using fractionated lignin and pMDI at an NCO/OH mole ratio of 0.3. The ratio of NCO/OH was calculated by dividing the mole of NCO of pMDI and the mole of phenolic OH in lignin. The fractionated lignin was dissolved in a NaOH 20% solution with a ratio of 1:10 (*w*/*v*). A solution of pMDI in acetone (8% *w*/*v*) was added to the lignin fractions. The mixture was mechanically stirred with a stirring speed of 500 rpm for 30 min for the polymerization reaction. The obtained Bio-PU resin was stored in vials before the characterization and impregnation of ramie fibers. The lignin-based Bio-PU formation reaction scheme is shown in Figure 2.

### 2.5. Impregnation of Ramie Fiber with Bio-PU

The impregnation of ramie fibers was carried out in a 1 L vacuum chamber with a vacuum pump (VC0918SS, VacuumChambers.eu, Białystok, Poland) according to the published work [44]. The initial weight of ramie fiber was recorded before vacuum impregnation. Five grams of ramie fibers were immersed in 50 mL of Bio-PU resin and impregnated at 25 ± 2 °C under the pressure of 50 kPa for 30, 60, and 90 min. The impregnated fiber was then dried in an oven at 60 °C for 24 h. Dried ramie fibers were weighed to determine the weight gain after impregnation. The weight gain (%) was calculated by dividing the mass of ramie fibers after impregnation by the initial mass of ramie fibers. The impregnated ramie fibers were then stored in a zip-lock plastic bag for further testing.

### 2.6. Characterization of Lignin-Based Bio-PU Resin

Functional groups of isolated lignin, fractionated lignin, and lignin-based Bio-PU resin were investigated using Fourier-transform infrared spectroscopy (FTIR) (SpectrumTwo, PerkinElmer, Waltham, MA, USA) by applying the universal attenuated total reflectance (UATR) method. The average accumulation was recorded as 16 scans at a resolution of 4 cm^−1^ with wavenumber in a range of 4000–400 cm^−1^ at 23 ± 2 °C.

Thermal properties of isolated lignin, fractionated lignin, and lignin-based Bio-PU were analyzed using Differential Scanning Calorimetry (DSC4000, PerkinElmer, Waltham, MA, USA). Around 4 mg of the sample was weighed in a standard aluminum pan (40 μL). The samples were heated in a nitrogen atmosphere with a flow rate of 20 mL/min and temperatures ranging from −50 to 350 °C with a heating rate of 10 °C/min. The *Tp*_1_ and *Tp*_2_ values were calculated automatically using Pyris 11 software (Version 11.1.1.0492, PerkinElmer, Shelton, CT, USA).

Thermogravimetric analysis (TGA) was performed using the TGA instrument (TGA 4000, Perkin Elmer, Waltham, MA, USA). Around 20 mg of the sample was weighed in a standard ceramic crucible and heated in a nitrogen atmosphere with a flow rate of 20 mL/min. The temperature used in heating ranged from 25 to 750 °C with a heating rate of 10 °C/min. The percent of weight loss, weight loss rate, and residue were analyzed with the help of Pyris software.

The analysis of lignin components was conducted using pyrolysis-gas-chromatography-mass-spectroscopy (Py-GCMS, Shimadzu, Kyoto, Japan). Lignin samples of 500–600 μg were put into the SF PYI-EC50F eco-cup, covered with glass wool, and Py-GCMS analyzed the samples. The eco-cup was pyrolyzed at 500 °C for 0.1 min using multi-shot pyrolysis (EGA/PY-3030D) connected to the GC/MS QP-2020 NX system (Shimadzu, Kyoto, Japan) and equipped with an SH-Rxi-5Sil column MS, which had a film thickness of 30 mm × 0.25 mm id 0.25 μm, 70 eV electrons, and helium as a carrier gas. The pressure used was 20.0 kPa (15.9 mL/min, column flow 0.61 mL/min). The temperature profile for GC was 50 °C, which was left for 1 min and then the temperature was increased to 280 °C with a heating rate of 5 °C/min, and the temperature was held at 280 °C for 13 min. Pyrolysis products were identified by comparing the retention time and mass spectrum data in the 2017 NIST LIBRARY program.

Rheological properties of lignin-based Bio-PU resin were investigated using a rotational rheometer (RheolabQC, AntonPaar, Graz, Austria). The measurement was conducted at room temperature using a concentric cylinder coupled with spindle No. 27. The viscosity and cohesion of lignin-based Bio-PU resin were obtained directly after the measurement.

### 2.7. Evaluation of Ramie Fibers Properties

FTIR, DSC, and TGA were performed as described in Section 2.6 to investigate the properties of ramie fibers before and after impregnation.

Micro confocal Raman hyperspectral imaging spectrometer (LabRAM HR Evolution, Horiba, Kyoto, Japan) was used to confirm the formation of urethane linkages on ramie fiber after impregnation. Ramie fiber before and after impregnation was put on the glass slide, and the image was captured using an NIR objective lens at 100-times magnification. The sample image then was bombarded with a laser at a wavelength of 785 nm using 600 grating. The measurement was performed at a Raman shift of 200–4000 cm^−1^, an acquisition time of 10 s, and accumulations of 10 at room temperature.

The determination of mechanical properties of ramie fiber was carried out in accordance with the ASTM D3379-75 standard using a universal testing machine (UTM 5kN, Shimadzu, Kyoto, Japan) [45]. The specimen used is a single fiber separated by strand bonds. The specimen length was 20–30 mm, and the total fiber length was about three times the length of the specimen. The specimens were tested at a temperature of 23 °C. The tensile strength and elastic modulus of ramie fibers before and after impregnation were calculated according to the ASTM D3379-75 [45].

## 3. Results and Discussion

### 3.1. Basic Properties of Isolated Lignin

Black liquor used in this study was obtained by the kraft pulping process of the pulp and paper industry. The characteristics of black liquor and isolated lignin are shown in Table 1. The yield of isolated lignin obtained was 35.88%, which is lower than the one-stage lignin precipitation by Hermiati et al. [38], namely 45.76%. The yield of isolated lignin is affected by the lignin content in black liquor from the kraft pulping process and the precipitation process during isolation. A complete precipitation reaction will produce a higher lignin yield.

The moisture content of isolated lignin (L-isolated) was 5.07%. The moisture content of lignin was relatively low compared to the published work [46], which was 8.05%. Ash content is the residual content of combustion of inorganic materials found in lignin. The low ash content of 0.31% indicated that the three-stage decantation process to obtain the lignin in this study produced fewer impurities compared to other studies that used a one-stage decantation process and obtained an ash content of 8.25–19.19% [38,47].

**Table 1:** Characteristics of lignin isolated from black liquor.

Analysis Parameters	Value	References
Moisture Content of Black Liquor (%)	27.81 ± 1.11	10.00 [48]
Solid Content of Black Liquor (%)	76.79 ± 0.64	65.00–85.00 [49,50]
pH of Black Liquor	12.14 ± 0.02	12.00–13.00 [51,52]
Yield of Lignin (%)	35.88 ± 1.81	45.76 [38]
Moisture Content of Lignin (%)	5.07 ± 0.71	8.05 [46]
Ash Content of Lignin (%)	0.31 ± 0.19	8.25–19.19 [38,47]
Acid-insoluble Lignin (AIL) (%)	82.54 ± 0.96	53.08 [38]
Acid-soluble Lignin (ASL) (%)	12.77 ± 0.67	7.26 [38]
Purity Levels of Lignin (%)	95.32 ± 0.61	60.34 [38]

The determination of lignin purity is based on AIL and ASL. As presented in Table 1, the AIL content was 82.54% and ASL content was 12.77%. The high AIL and ASL values determined can be attributed to the three-stage decantation process during isolation. The decantation process involved washing with distilled water to remove impurities and other substances, thus increasing the total lignin generated from the isolation process. The level of purity of the isolated lignin was relatively high, namely 95.32%. This result was higher than the published works that used a similar source of black liquor and acid precipitation methods [38], calculating around 60.34% of lignin purity. The difference in the level of lignin purity can be attributed to the differences in mineral content in lignin isolates.

The yield of dissolved fractionation is shown in Table 2. The yield of lignin fractionated with MeOH (L-MeOH) was 71.25% and lignin fractionated with acetone (L-Ac) was 70.57%. The results are in agreement with the published work [53], where the highest solubility of fractionated technical lignin was observed in methanol and acetone solvents of 70–80%. The solubility of this lignin in organic solvents depends on the type of lignin, aliphatic hydroxyl number, and molecular weight [54].

Total phenolic hydroxyl groups of fractionated lignin were determined using the UV method. The UV method is a fast and easy way of measuring phenolic hydroxyl groups by giving the total number of hydroxyl (OH) groups without any structural specifications [43]. The total phenolic OH group of L-Standard, L-Isolated, and fractionated lignin are shown in Table 2. The highest total OH group was found in the L-Standard (8.109%), and the lowest was obtained in the L-MeOH (7.399%). The isolated lignin contains high phenolic hydroxyl groups and a condensed structure due to the kraft pulping process [39]. The OH group value was further used in the formulation of lignin-based Bio-PU resin with an NCO/OH mole ratio of 0.3.

### 3.2. Characterizations of Lignin

#### 3.2.1. FTIR Analysis

**Figure 3:**
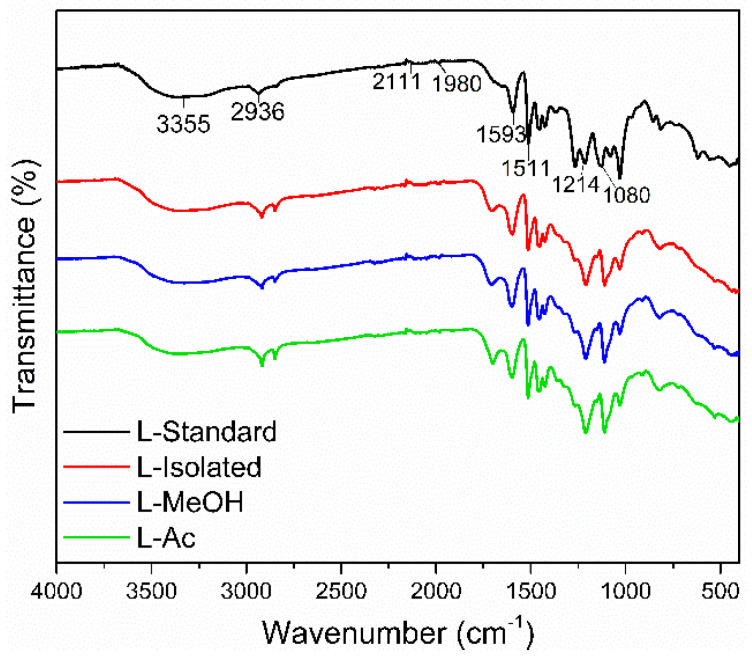
Typical FTIR spectra of lignin before and after fractionation.

FTIR spectra of isolated lignin and fractionated lignin are shown in Figure 3. Standard lignin was used as a comparison. The isolated lignin and fractionated lignin were similar to standard lignin in terms of functional groups. However, there were different intensities at specific wavenumbers of isolated lignin and fractionated lignin.

The broad absorption band at 3550–3200 cm^−1^ is the O-H stretching vibration in phenolic and aliphatic O-H groups. The peaks were detected at 3355 cm^−1^ for standard lignin, 3347 cm^−1^ for isolated lignin, and 3327 cm^−1^, and 3356.47 cm^−1^ for L-MeOH and L-Ac, respectively. The wavenumber at 2936–2917 cm^−1^ shows typical C-H stretching of CH_3_ and CH_2_, 1710–1698 cm^−1^ was assigned to C=O stretching from conjugate acid, 1601–1593 cm^−1^ was attributed to C=O stretching vibrations from skeletal aromatic, and 1514–1511 cm^−1^ represented the C-C stretching of the aromatic ring. The absorption bands at 1605–1600 cm^−1^ and 1515–1505 cm^−1^ were assigned to the aromatic ring vibration of the phenyl-propane (C9) skeleton [47]. The peaks at 1470–1460 cm^−1^ indicated the presence of C-H deformation (asymmetrical) of methyl, methylene, and methoxyl groups, which indicated the aromatic structure of lignin did not change after the isolation and fractionation process.

Standard lignin showed strong absorption bands at 1269 cm^−1^ and 1210–1220 cm^−1^, assigned to the C-O group of guaiacyl, phenolic O-H, and ether in syringyl and guaiacyl. Meanwhile, isolated lignin and fractionated lignin have a strong absorption band at 1080–1030 cm^−1^, indicating a C-O group in syringyl and guaiacyl. It is suspected that the standard lignin used is a guaiacyl lignin derived from softwood, which has a higher intensity at a wavenumber of 1269 cm^−1^. Isolated lignin and fractionated lignin were thought to be syringyl-guaiacyl lignin derived from hardwood and have a higher intensity at a wavenumber of 1030 cm^−1^ [55]. This study showed that isolated lignin from black liquor and fractionated lignin did not damage the structure of lignin.

#### 3.2.2. DSC Analysis

**Figure 4:**
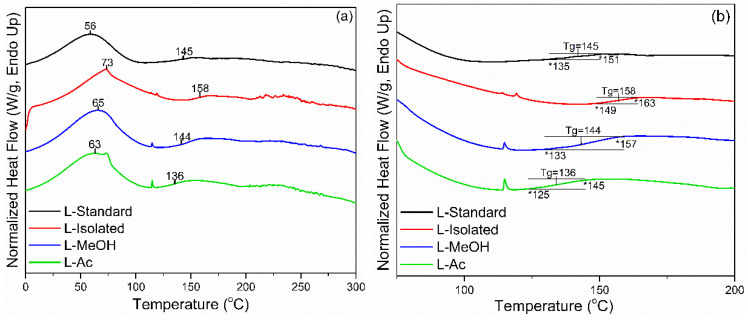
DSC thermograms of lignin before and after fractionation: (**a**) full thermogram, (**b**) enlarged thermogram.

Thermal properties of isolated and fractionated lignin were analyzed using DSC analysis. Standard lignin was used as a comparison. In Figure 4, two endothermic peaks were detected during the heating process of lignin. The initial peak endothermic reaction (*Tp*_1_) appeared at 56 °C for L-standard and 73 °C for L-isolated. Meanwhile, *Tp*_1_ for fractionated lignin was in the range of 63–65 °C. The initial endothermic reaction is the beginning of the process to evaporate the water contained in lignin. The higher *Tp*_1_ of isolated and fractionated lignin indicated that lignin had more water than standard lignin. The second endothermic reaction (*Tp*_2_) is the final part of the endothermic process that changes the lignin structure and reduces stiffness (plasticization). This reaction is also known as the glass transition (*Tg*), which is the inflection point of the heat flow change of the heating cycle [56]. The *Tg* of lignin is generally in the range of 100–180 °C, and *Tg* from standard lignin, isolated lignin, and fractionated lignin were in the range of 136–158 °C.

#### 3.2.3. TGA Analysis

**Figure 5:**
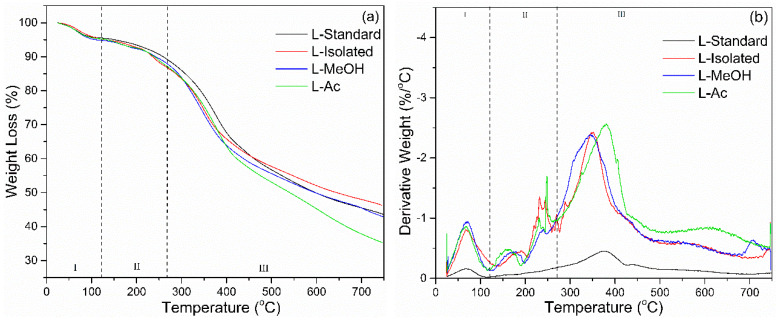
Thermal behavior of lignin before and after fractionation: (**a**) TGA, (**b**) DTG.

The thermal degradation of lignin was determined by TGA-DTG analysis. The results of the TGA-DTG analysis are shown in Figure 5. There are three stages of lignin degradation based on the TGA-DTG analysis. The first stage occurs at a temperature of 25–100 °C, which causes lignin to lose weight due to water evaporation or reduction in moisture. At this stage, the derivative weight loss was around 0.2–1.0%/°C. The next step is weight reduction due to carbohydrate degradation, which occurs at a temperature of 120–250 °C. In this state, lignin lost weight up to 10% with a derivative weight loss of around 0.5%/°C. The main lignin degradation occurred at the broadest temperature range from 200 to 480 °C. Based on the DTG curve, the most significant lignin degradation occurs at a temperature range of 370 °C with a derivative weight loss reaching an average of 2.5%/°C.

Lignin loses ~50% of its weight in the temperature interval of 400–650 °C (Table 3). The heterogeneous structure of lignins with a non-repetitive combination of carbon–carbon and ether linkages and with a broad molecular mass distribution results in the facile release of considerable amounts of volatile products. At temperatures above 500 °C, lignin degradation occurs, associated with the decomposition of the aromatic ring. At a temperature of 750 °C, the final percentage of residual lignin combustion was in the range of 31–46%. Isolated lignin had the most percentage of lignin residue (46.25%) and L-Ac had the smallest residual percentage (35.29%). Isolated lignin has better thermal stability compared to fractionated lignin. Fractionation of lignin broke down lignin macromolecules into smaller molecules, which resulted in lower thermal stability. It has been reported that lignin is more thermally stable, so it can be used as a synthesis agent for polyurethane and phenol-formaldehyde resin in high-temperature applications [57].

#### 3.2.4. Py-GCMS Analysis

**Figure 6:**
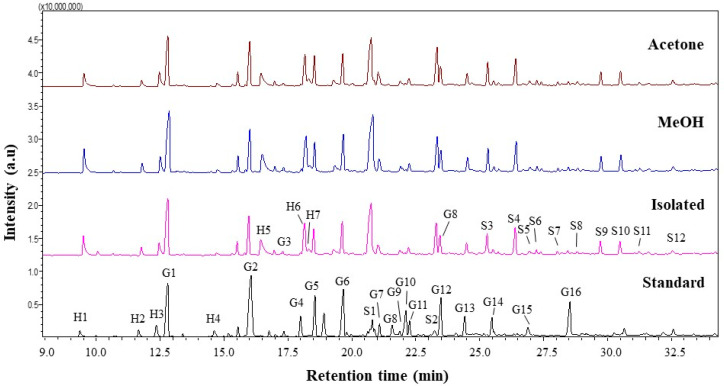
Py-GCMS chromatograms of lignin before and after fractionation.

Lignin is a complex polymer synthesized from three hydroxyl alcohols that differ in degree of methoxylation, namely p-coumaryl, coniferyl, and synapyl alcohols. Each monolignol produces a different type of lignin unit called p-hydroxyphenyl (H), guaiacyl (G), and syringyl (S). Pyrolysis gas chromatography/mass spectrometry (Py-GCMS) was performed as analysis to determine the lignin composition contained in isolated lignin and fractionated lignin. Pyrolysis is based on thermal fragmentation at high temperatures up to 500 °C and in an oxygen-free environment. The results of the Py-GCMS chromatogram of isolated lignin and fractionated lignin are shown in Figure 6. The results showed that isolated lignin and fractionated lignin produced more syringyl than guaiacyl and hydroxyphenyl, which were recorded at retention times of 21 min to 32.5 min. Meanwhile, standard lignin produced more guaiacyl, which became visible at retention times of 12.5 min to 28.5 min (Figure 6). Based on the result, the S/G ratio of isolated lignin is 0.96. Meanwhile, the fractionated lignin had a higher S/G ratio, which was 0.99 for L-MeOH and 1.01 for L-Ac. A similar trend was found for the S/GH ratio, where fractionated lignin had a greater S/G/H ratio than the isolated lignin (Table 4). The prominent peaks in the lignin chromatogram obtained were Guaiacol (G1), Syringol (S1), Guaiacol. 4-methyl (G2), Syringol-4-methyl (S2), and Guaiacol. 4-vinyl (G5) [58].

### 3.3. Properties of Bio-Polyurethane Resin

**Figure 7:**
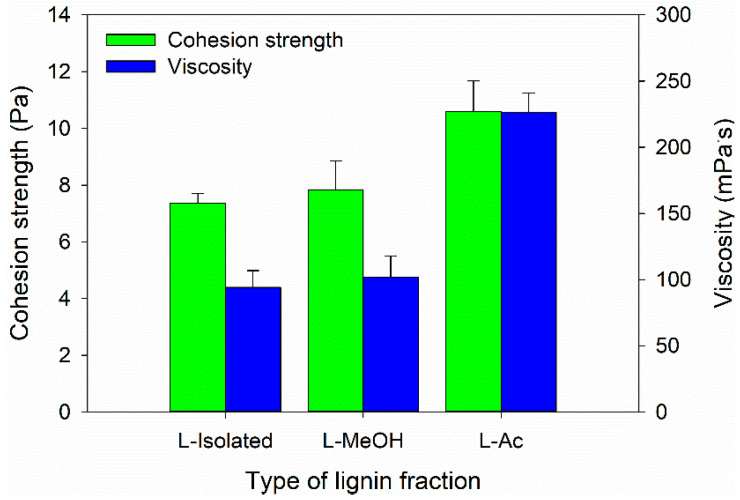
Viscosity and cohesion strength of Bio-PU from fractionated lignin.

In this study, the Bio-PU resin was designed as an impregnation material to improve the characteristics of ramie fiber. Therefore, the Bio-PU resin must have a low viscosity to be impregnated into the ramie fiber [59]. The viscosity and cohesion strength of Bio-PU resin was measured at 25 °C (Figure 7). The lowest viscosity value of 77.02 mPa·s was determined for Bio-PU L-Isolated, followed by Bio-PU L-MeOH and Bio-PU L-Ac, which had values of 100.71 and 223.58 mPa·s, respectively (Figure 7). The cohesion strength followed the results of viscosity, where the cohesion strength increased with greater viscosity of Bio-PU resin. Lower viscosity can increase the ability of Bio-PU resin to be impregnated and absorbed by ramie fiber. Viscosity is related to hydrogen bonds between isocyanate and polyol molecules (NCO/OH molar ratio). An increase in the NCO/OH ratio will result in more urethane bonds forming hard segments in Bio-PU and produce high viscosity [59,60].

**Figure 8:**
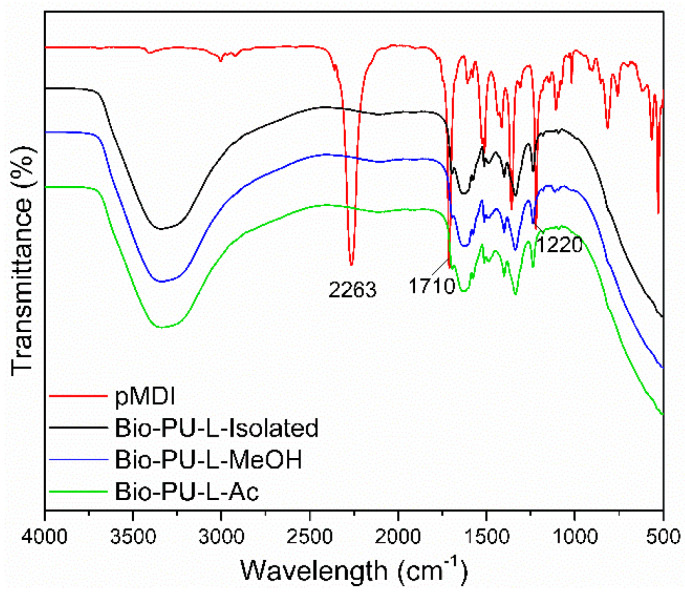
ATR-FTIR spectra of Bio-PU resin derived from fractionated lignin.

The functional groups of Bio-PU resin are shown in Figure 8. A typical strong peak at the wavenumber of 2263 cm^−1^ indicated an isocyanate group (N=C=O) of pMDI. In the Bio-PU resin (L-isolated, L-MeOH, and L-Ac), no absorption of the N=C=O group was found, indicating that the N=C=O group reacted completely with the OH group of lignin during the preparation of PU resin. It has been reported that the isocyanate group derived from pMDI will react with the hydroxyl group of the polyol [61]. The reaction between fractionated lignin and pMDI in the formation of Bio-PU resin produces a specific functional group, namely the urethane group (R-NH-C=O-R) [62].

Bio-PU resin showed broad peaks at wavenumbers 3400–3200 cm^−1^, indicating the formation of N-H groups in the aliphatic primary amine structure. In addition, this broad peak also overlapped with the -OH groups from the lignin sample and aqueous NaOH for lignin dissolution. The urethane bond formed from the lignin phenolic OH group and isocyanate (NCO group) from pMDI [63]. Another characteristic band in the formation of the Bio-PU resin at 1710–1685 cm^−1^ revealed the formation of C=O stretching from the urethane bond (R-NH-C=O-R), and the wavenumber of 1250–1020 cm^−1^ showed C-N stretching. It is indicated that the Bio-PU resin was successfully prepared from fractionated lignin (L-MeOH and L-Ac) as an alternative polyol.

**Figure 9:**
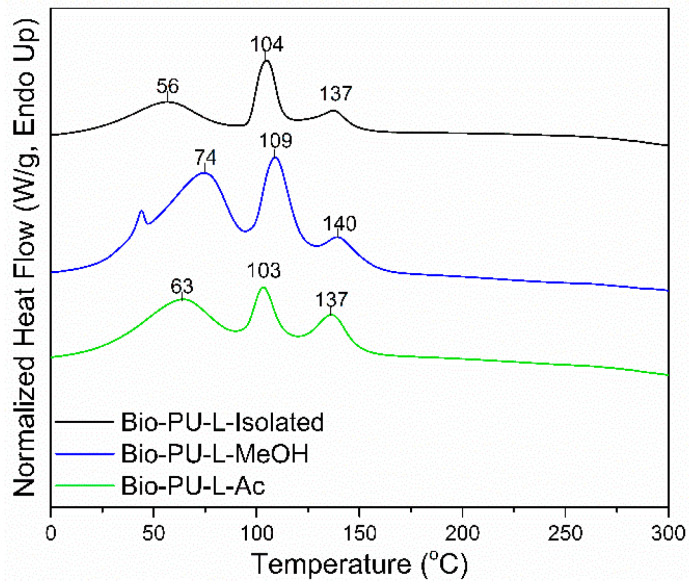
DSC thermograms of Bio-PU resin derived from fractionated lignin.

Thermal properties of the Bio-PU resin were determined based on the results of the DSC analysis shown in Figure 9. The DSC thermograms show three-step endothermic reactions in the Bio-PU resin. The *Tp*_1_ of Bio-PU (L-Isolated, L-MeOH, and L-Ac) ranged 56–63 °C, the *Tp*_2_ ranged 103–109 °C, and the *Tp*_3_ or *Tg* ranged 137–140 °C. The *Tp*_1_ corresponded to the initial moisture evaporation in Bio-PU resin and the evaporation peak at *Tp*_2_. The *Tg* value represents the polymer transition at a specific temperature [64]. The *Tg* of Bio-PU L-MeOH was 140 °C; meanwhile, the *Tg* of Bio-PU L-isolated and L-Ac was 137 °C. High *Tg* produces hard segments in Bio-PU due to the formation of urethane. Inter-urethane hydrogen bonds play an essential role in the stability of Bio-PU resin. The structure of the hard segment is more influential in thermal degradation than the soft segment [65].

**Figure 10:**
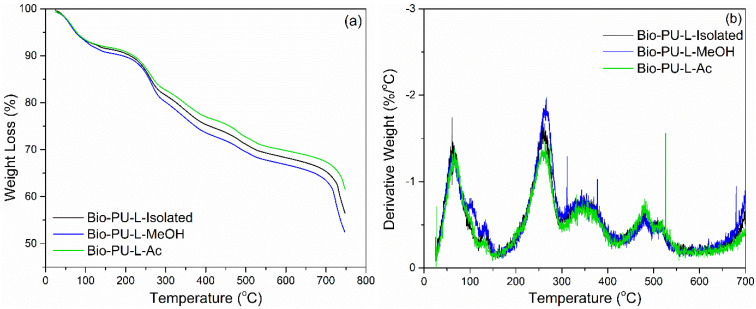
Thermal degradation of Bio-PU resin derived from lignin: (**a**) TGA, (**b**) DTG.

Thermogravimetric analysis was performed to evaluate the thermal degradation of lignin-based Bio-PU resin. TGA-DTG thermogram is shown in Figure 10. The initial weight loss occurred at a temperature of 60–110 °C, caused by water evaporation and evaporation of chemicals in unreacted lignin during the polymerization process [44,61]. Lignin-based Bio-PU lost 10% of the initial weight with a derivative weight loss of 1.5%/°C at a temperature of 80 °C. There was significant weight loss at temperatures above 160 °C up to 300 °C. The degradation peak occurred at a temperature of 250 °C, which indicated the initial decomposition of the urethane bond with a derivative weight loss of 2.0%/°C. Further degradation occurred at ~350 °C, which is oxidative degradation by the urethane group with a derivative weight loss of 0.75%/°C [66]. At a temperature of 450–600 °C, both the aromatic ring decomposition of lignin and the primary oxidative decomposition of lignin occurred [66,67].

The weight residue after heating at 750 °C of each Bio-PU resin (L-Isolated, L-MeOH, and L-Ac) was in the range of 52–61% (Figure 10). Bio-PU L-Ac had the highest weight residue of 61.55 and bio-PU L-MeOH had the lowest weight residue of 52.76%. The bio-PU L-Ac showed lower thermal degradation compared to Bio-PU from L-Isolated and L-MeOH due to crosslinking the lignin macromonomers. The weight loss of Bio-PU from each fractionation of lignin was 25% at 300–400 °C during degradation of the urethane group. The weight loss of the fractionated lignin-based Bio-PU resin in this study was not more than 50%. Meanwhile, rigid or semi-rigid polyurethane resin has a percentage of weight loss at 340–525 °C of 62–75% [68]. This study showed that lignin fractionation using Acetone (Ac) could produce lignin-based Bio-PU resin with lower thermal degradation than isolated lignin.

### 3.4. Properties of Ramie Fiber Impregnated with Bio-PU Resin

The impregnation of ramie fiber with lignin-based Bio-PU was aimed at improving ramie fiber’s thermal stability as a functional material. The weight gain of ramie fiber after impregnation was calculated as a way to investigate the influence of the impregnation process on ramie fiber. The weight gain of ramie fiber after impregnation is shown in Table 5. In general, the weight gain increased with longer impregnation time. Impregnation for 90 min resulted in more significant weight gain for each type of lignin-based Bio-PU used, followed by impregnation times of 60 and 30 min. Ramie impregnated with Bio-PU derived from L-isolated had the highest weight gain compared to Bio-PU L-MeOH and L-Ac. This could be due to the viscosity of Bio-PU L-isolated being lower than that of L-MeOH and L-Ac (Figure 6). The lower viscosity of Bio-PU will make it easier for the solution to be impregnated into the fiber. Ramie fiber has a fiber diameter of 40–60 µm [69]. This could facilitate the absorption of the Bio-PU solution and increase weight gain after Bio-PU impregnation into the ramie fiber.

**Figure 11:**
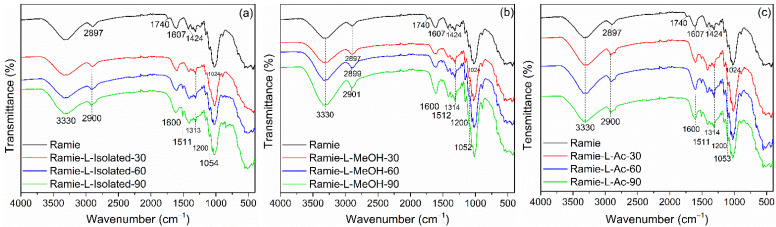
FTIR Spectra of impregnated ramie fibers: (**a**) Ramie L-Isolated, (**b**) Ramie L-MeOH, and (**c**) Ramie L-Ac.

The FTIR spectra of the impregnated ramie fiber investigated in this study are shown in Figure 11. In non-impregnated ramie fiber, there were six prominent peaks. Firstly, a wavenumber of 3330 cm^−1^ represented OH groups from cellulose, hemicellulose, and lignin that arrange the ramie fiber [70]. A wavenumber of 2897 cm^−1^ described C-H vibration carbohydrates [44]. A wavenumber of 1740 cm^−1^ indicated the presence of C=O vibration in the ester, carboxylate groups, and hemicellulose of ramie fiber [70,71,72]. Wavenumbers of 1607 cm^−1^, 1424 cm^−1^, and 1024 cm^−1^ showed the C-H stretching of carbohydrates, C-O stretching of cellulose, C-O vibrations, and O-H vibrations of cellulose, respectively [44,72].

The functional groups of ramie fiber after impregnation were also observed using FTIR. A peak similar to the original ramie at a wavenumber of 3330 cm^−1^ indicated O-H vibration. The peak of ramie fiber at 2897 cm^−1^ shifted to 2900 cm^−1^, which showed N-H bending and -CH_2_ stretching of bio-PU in impregnated ramie fiber with Bio-PU resins. The wavenumber of 1740 cm^−1^ was not found in ramie fiber after impregnation. It can be assumed that there was a reaction between ramie fiber and Bio-PU resins, and the formation of a peak at 1600 cm^−1^ indicates the formation of C=O urethane from Bio-PU [44]. The wavenumber of 1515–1310 cm^−1^ represented the C-N stretching group from primary and secondary amides of bio-PU resin in impregnated ramie fiber. Furthermore, the wavenumbers 1200 cm^−1^ and 1050 cm^−1^ showed C=O vibrations of Bio-PU and C-O-C ether linkages, respectively [19], which demonstrates that lignin-based Bio-PU successfully impregnated the ramie fiber.

**Figure 12:**
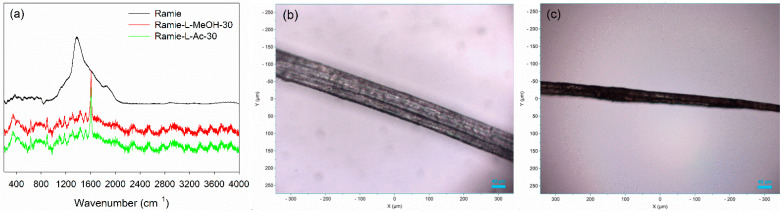
Micro Confocal Raman analysis of ramie fiber: (**a**) Typical Raman spectra of ramie fiber, (**b**) image of ramie fiber before impregnation at 10× magnification, and (**c**) image of ramie fiber after impregnation at 10× magnification.

**Figure 13:**
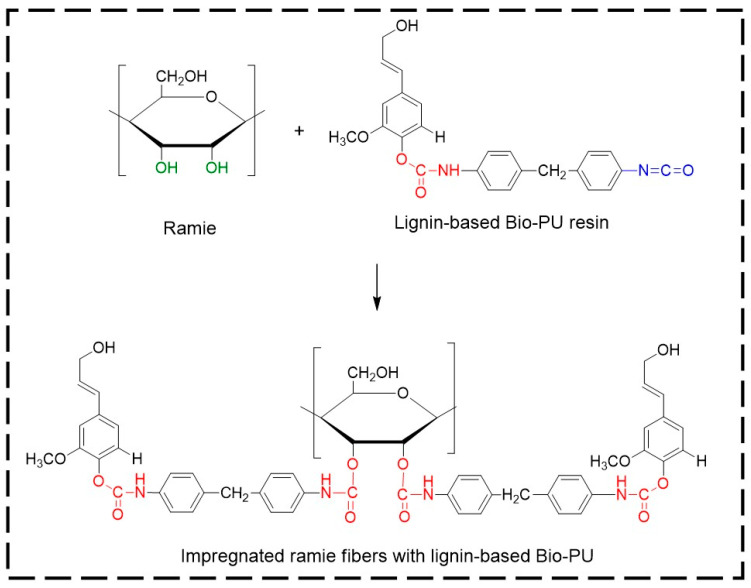
The possible scheme reaction of ramie fibers with lignin-based Bio-PU resin.

Micro Confocal Raman analysis was performed to investigate the formation of urethane linkages in ramie fiber after impregnation with lignin-based Bio-PU resin. As depicted in Figure 12a, the original ramie fiber had a typical broad peak at 1350–1400 cm^−1^, which indicated the presence of cellulosic materials. After impregnation with Bio-PU resin, the Raman spectra of ramie fiber remarkably changed due to the presence of a strong peak of urethane linkages at 1614 cm^−1^. This result confirmed that Bio-PU resin impregnated and covered the ramie fiber. The images of ramie fiber before and after impregnation are presented in Figure 12b,c. The results showed that impregnation of Bio-PU into ramie fiber altered the color of ramie fiber to a light brown. Based on the result of FTIR and Micro Confocal Raman Hyperspectral Spectroscopies, the possible reaction of ramie fiber with lignin-based Bio-PU resin is illustrated in Figure 13.

**Figure 14:**
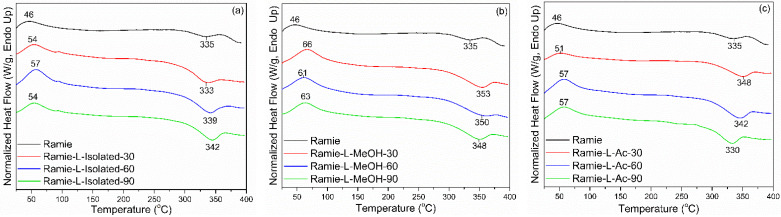
DSC analysis of impregnated ramie fibers: (**a**) Ramie L-Isolated, (**b**) Ramie L-MeOH, and (**c**) Ramie L-Ac.

DSC analysis detected two thermal events on ramie fiber before and after impregnation, namely an endothermic reaction and an exothermic reaction (Figure 14). The endothermic reaction is a heat absorption reaction by the ramie fiber, while the exothermic reaction is a heat release reaction by the ramie fiber [73]. The peak of the endothermic reaction (*Tp*_1_) in the original ramie fiber occurred at a temperature of 46 °C, which is the process of evaporation of water. In general, impregnation resulted in a slightly higher *Tp*_1_ value. Ramie fiber impregnated with L-Isolated and L-Ac had a lower *Tp*_1_ value than that of ramie impregnated with L-MeOH. The difference in *Tp*_1_ value between natural ramie and impregnated ramie is influenced by differences in fiber moisture content. The moisture content of natural ramie is 5.15%, and the moisture content of impregnated ramie is between 6 and 10%. No significant influence of increasing the impregnation time on the *Tp*_1_ value of ramie impregnated with lignin-based Bio-PU resin was observed.

The exothermic reaction in ramie fiber indicates the formation of solid residues due to hemicellulose decomposition and lignin degradation [74]. The peak of the exothermic reaction (*Tp*_2_) generally occurred after the heating temperature reached 300 °C. In non-treated ramie, the *Tp*_2_ value was around 335 °C. Impregnation resulted in a slightly higher *Tp*_2_ value. Ramie fiber impregnated with L-Isolated and L-Ac had a lower *Tp*_2_ value than that of ramie impregnated with L-MeOH. The increase in *Tp*_2_ value in impregnated ramie compared to original ramie indicates that ramie has more heat-resistant properties after impregnation. Ramie fiber impregnated at 90 min for all types of Bio-PU generally had an increasing *Tp*_2_ value compared to original ramie.

**Figure 15:**
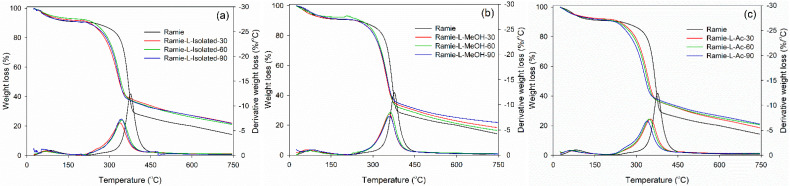
Thermal stability of impregnated ramie fibers determined with TGA-DTG: (**a**) Ramie L-Isolated, (**b**) Ramie L-MeOH, and (**c**) Ramie L-Ac.

Thermal degradation of ramie fiber before and after impregnation with lignin-based Bio-PU resin was monitored using TGA-DTG analysis. As displayed in Figure 15, the initial stage of the degradation process occurred at a temperature of 25–100 °C, which is attributed to the release of water contained in ramie fiber and the degradation of low-molecular-weight components. At this stage, the ramie fiber lost 5% weight with a derivative weight loss of around 1.0–2.0%/°C. Significant weight loss was observed after heating at 250–450 °C, which caused the ramie fiber to experience an extreme weight loss of around 75% at a derivative weight loss of 13%/°C. The results showed that the impregnation of ramie fiber with Bio-PU remarkably enhanced the thermal stability by reducing the weight loss value to 50% with a lower derivative weight loss of 6.0–7.0%/°C. At a temperature of 200–400 °C, a complex reaction occurs from the degradation of the constituent components of lignocellulose fibers, such as cellulose, hemicellulose, and lignin [75]. Hemicellulose begins to degrade at a temperature of 200–290 °C, at a temperature of 240–350 °C, cellulose degradation occurs, and lignin degradation begins at a temperature of 280–500 °C. Several related studies also stated that the degradation of lignin, cellulose, and hemicellulose in ramie fiber occurred at a temperature of 300–400 °C, whereas at a temperature of 320–400 °C, the cellulose glycosidic bond was broken, and the decomposition of the lignin structure by a high molecular weight followed at a temperature of 360–750 °C [44,72,76].

The impregnated ramie fiber lost 75% of its weight at a temperature of 550–660 °C, while the non-impregnated ramie fiber experienced a similar weight loss at 469 °C. This can be attributed to the occurrence of lignin degradation at that temperature. Maximum decomposition occurred at a temperature of 300–400 °C, and has also been noted that lignin degradation in ramie fiber is the limit for determining thermal stability [76]. This study showed that lignin-based Bio-PU can increase the thermal stability of ramie fiber so that it is better than natural ramie. The impregnation time affects the thermal degradation of the ramie fiber remarkably. The longer the impregnation time, the lower the thermal degradation of the impregnated fiber. The weight residue of the original ramie fiber was 14.3%. Meanwhile, impregnated ramie has a higher residue weight, at 18.4–21.7%. An impregnation time of 90 min produced lower thermal degradation of ramie fiber with greater weight residue than those at 30 and 60 min. The lowest thermal degradation was obtained in both ramie fiber impregnated with L-MeOH and L-Ac for 90 min with an average weight residue of around 21.7%.

**Figure 16:**
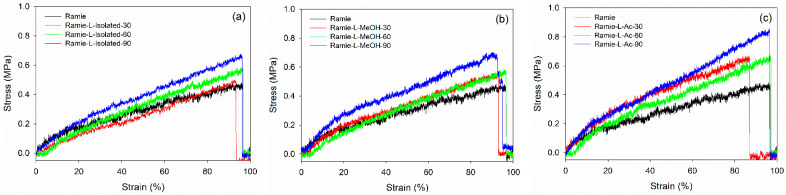
Typical stress–strain curve of ramie fibers before and after impregnation: (**a**) Ramie L-Isolated, (**b**) Ramie L-MeOH, and (**c**) Ramie L-Ac.

Mechanical properties are essential characteristics for ramie fiber as a textile and composites material. A graphical representation of the stress-–strain curves of ramie fiber impregnated with different lignin-based Bio-PU resins for different impregnation times is presented in Figure 16. The results showed that increasing the impregnation time generally enhanced the maximum stress. Ramie impregnated with Bio-PU L-Ac showed the highest maximum stress after 90 min of impregnation, followed by Bio-PU L-isolated and L-MeOH. The lowest stress was obtained in the original ramie fiber. This indicated that the impregnation of Bio-PU resin into ramie fiber could enhance the mechanical properties of the fiber. The fiber deformation can be divided into the following three stages: (i) A linear part related to the deformation of the fiber cell wall; (ii) a non-linear part interpreted as a thicker cell wall deformation (S2) and known as elasto-visco-plastic; and (iii) a final linear section, which is the elastic response aligned with the tensile strain [33].

The values obtained for the modulus of elasticity (MOE) and tensile strength of non-impregnated and impregnated ramie fibers are presented in Table 6. In general, the mechanical properties of impregnated ramie fibers increased compared to the original ramie. The tensile strength of the original ramie was 397.72 MPa with an MOE value of 10.45 GPa. The tensile strength of impregnated ramie was in the range of 397.72–648.48 MPa with MOE values ranging from 13.06 to 31.10 GPa. Increasing the impregnation time generally increased the elastic modulus and tensile strength of ramie fiber. Ramie fiber impregnated with Bio-PU L-isolated for 90 min resulted in the highest MOE value of 31.10 GPa. Meanwhile, ramie impregnated with Bio-PU L-Ac for 90 min had the highest tensile strength of around 577.61 MPa. Several factors besides chemical modification affected the mechanical properties of ramie fiber, namely parameters related to relative environmental humidity, fiber length, fiber microstructure, moisture content and drying, and fiber diameter [77,78].

## 4. Conclusions

This study demonstrated the potential use of lignin derived from black liquor as a pre-polymer of bio-polyurethane (Bio-PU) resin and its application for the modification of ramie fiber. The isolated lignin from black liquor was fractionated using MeOH and Ac. The isolated and fractionated lignin then reacted with pMDI to form Bio-PU resin with an NCO/OH ratio of 0.3. FTIR results showed that the reaction between -OH of lignin and -NCO of pMDI formed an absorption band at a wavenumber of 1605 cm^−1^, which was urethane linkages (R-NH-C=O-R) of Bio-PU resin. The Bio-PU L-Isolated has a lower viscosity than fractionated lignin. The thermal properties and thermal stability of Bio-PU L-Ac were better than L-isolated and L-MeOH. Impregnation of Bio-PU into ramie fiber resulted in weight gain varying from 6% to 15%, where the value increased with longer impregnation time. The reaction between Bio-PU and ramie fiber formed C=O of the urethane group as confirmed by FTIR and Micro Confocal Raman Spectroscopies. This resulted in greater thermal properties and stability of ramie fiber after impregnation with the weight residue reaching 21.7%. The mechanical properties of ramie fiber also increased after impregnation with lignin-based Bio-PU, resulting in a modulus of elasticity of around 31 GPa for Ramie-L-isolated and tensile strength of around 577 MPa for Ramie-L-Ac. This study showed that lignin-based Bio-PU resin derived from isolated lignin and fractionated L-Ac could be used as a pre-polymer of Bio-PU resin for the modification of ramie fiber via impregnation. The enhanced thermal stability and mechanical properties of impregnated ramie fiber could increase the future potential for greater industrial application of ramie fiber as a sustainable and functional material.

## Figures and Tables

**Table 2:** The yield and total phenolic hydroxyl group of isolated and fractionated lignin.

Type of Lignin	Yield of Fractionated Lignin (%)	Total OH Group
L-Standard	-	8.109
L-Isolated	-	7.968
L-MeOH	71.25	7.399
L-Ac	70.57	7.645

**Table 3:** Degradation temperature and weight loss of lignin detected by TGA.

Type of Lignin	*T_WL_*_10%_ (°C)	*T_WL_*_25%_ (°C)	*T_WL_*_50%_ (°C)	WL (%)	Residue (%)
L-standard	259	366	597	56.49	43.51
L-isolated	235	347	644	53.75	46.25
L-MeOH	242	341	596	57.06	42.94
L-Ac	239	351	540	64.71	35.29

**Table 4:** Pyrolysis products of lignin assessed by Py-GCMS.

No	RT (min)	Pyrolysis Product	Origin	Lignin-Isolated	Fractionated Lignin	
					MeOH	Ac
1	9.50	Phenol	H	3.93	4.17	2.85
2	11.76	Phenol. 2-methyl-	H	1.22	1.35	1.35
3	12.46	Phenol. 4 methyl	H	2.7	2.9	3.13
4	12.80	Guaiacol	G	14.24	14.87	10.86
5	15.97	Guaiacol. 4-methyl	G	6.79	6.66	8.7
6	16.44	Catechol	H	4.65	5.04	4.07
7	18.16	Catechol. 3-methoxy	H	7.28	6.58	6.72
8	18.30	Catechol. 4 methyl	H	1.44	0.2	1.84
9	19.62	Guaiacol. 4-vinyl	G	5.58	5.61	5.23
10	20.76	Syringol	S	16.77	17.5	14.38
11	23.30	Syringol-4-methyl	S	6.24	6.3	8.36
LH (hydroxypenhyl)				21.22	20.24	19.96
LG (guaiacyl)				39.84	39.94	39.51
LS (syringyl)				38.52	39.44	39.9
S/G				0.9669	0.9875	1.0099
S/G/H				0.0456	0.0488	0.0506

**Table 5:** Weight gain of ramie fiber after impregnation with lignin-based Bio-PU at 30, 60, and 90 min.

Type	Weight Gain (%)		
	30 min	60 min	90 min
Ramie L-Isolated	12.38 ± 1.69	15.17 ± 0.36	15.93 ± 2.43
Ramie L-MEOH	6.25 ± 1.10	7.21 ± 3.17	8.62 ± 1.09
Ramie L-Ac	6.68 ± 0.74	8.26 ± 0.06	9.07 ± 0.62

**Table 6:** Modulus of elasticity (MOE) and tensile strength of ramie and impregnated ramie fibers at different impregnation times.

Type	Impregnation Time (min)	Modulus of Elasticity (GPa)	Std. Dev	Tensile Strength (MPa)	Std. Dev
Ramie	-	10.45	0.2402	397.72	58.26
Ramie-L-isolated	30	15.23	0.4169	441.19	226.37
	60	20.35	0.6712	497.22	91.62
	90	31.10	0.6705	574.11	133.97
Ramie-L-MeOH	30	13.06	0.4446	447.06	58.59
	60	11.98	0.2780	406.71	175.64
	90	17.86	0.5629	461.32	106.13
Ramie-L-Ac	30	16.78	0.2554	523.38	80.12
	60	19.81	0.2923	547.66	91.37
	90	21.99	0.5288	577.61	68.87

## Data Availability

The data presented in this study are available on request from the corresponding author.

## References

[1] Hwang J.Z., Wang S.C., Chen P.C., Huang C.Y., Yeh J.T., Chen K.N. (2012). A new UV-curable PU resin obtained through a nonisocyanate process and used as a hydrophilic textile treatment. J. Polym. Res..

[2] Zia K.M., Anjum S., Zuber M., Mujahid M., Jamil T. (2014). Synthesis and molecular characterization of chitosan based polyurethane elastomers using aromatic diisocyanate. Int. J. Biol. Macromol..

[3] Das A., Mahanwar P. (2020). A brief discussion on advances in polyurethane applications. Adv. Ind. Eng. Polym. Res..

[4] Chen W.-J., Wang S.-C., Chen P.-C., Chen T.-W., Chen K.-N. (2008). Hybridization of aqueous PU/epoxy resin via a dual self-curing process. J. Appl. Polym. Sci..

[5] Wang S.-C., Chen P.-C., Yeh J.-T., Chen K.-N. (2008). Curing reaction of amino-terminated aqueous-based polyurethane dispersions with triglycidyl-containing compound. J. Appl. Polym. Sci..

[6] Strakowska A., Członka S., Kairyte A. (2020). Rigid polyurethane foams reinforced with poss-impregnated sugar beet pulp filler. Materials.

[7] Uram K., Kurańska M., Andrzejewski J., Prociak A. (2021). Rigid Polyurethane Foams Modified with Biochar. Materials.

[8] Gama N.V., Ferreira A., Barros-Timmons A. (2018). Polyurethane foams: Past, present, and future. Materials.

[9] Kurańska M., Pinto J.A., Salach K., Barreiro M.F., Prociak A. (2020). Synthesis of thermal insulating polyurethane foams from lignin and rapeseed based polyols: A comparative study. Ind. Crops Prod..

[10] Hu S., Luo X., Li Y. (2014). Polyols and polyurethanes from the liquefaction of lignocellulosic biomass. ChemSusChem.

[11] Petrovic Z.S. (2008). Polyurethanes from vegetable oils. Polym. Rev..

[12] Pfister D.P., Xia Y., Larock R.C. (2011). Recent advances in vegetable oil-based polyurethanes. ChemSusChem.

[13] Lligadas G., Ronda J.C., Galiá M., Cádiz V. (2010). Plant oils as platform chemicals for polyurethane synthesis: Current state-of-the-art. Biomacromolecules.

[14] Ge X., Chang C., Zhang L., Cui S., Luo X., Hu S., Qin Y., Li Y. (2018). Conversion of Lignocellulosic Biomass into Platform Chemicals for Biobased Polyurethane Application.

[15] Thébault M., Pizzi A., Essawy H.A., Barhoum A., Van Assche G. (2015). Isocyanate free condensed tannin-based polyurethanes. Eur. Polym. J..

[16] Aristri M.A., Lubis M.A.R., Iswanto A.H., Fatriasari W., Sari R.K., Antov P., Gajtanska M., Papadopoulos A.N., Pizzi A. (2021). Bio-Based Polyurethane Resins Derived from Tannin: Source, Synthesis, Characterisation, and Application. Forests.

[17] Watanabe M., Kanaguri Y., Smith R.L. (2018). Hydrothermal separation of lignin from bark of Japanese cedar. J. Supercrit. Fluids.

[18] Tudor E.M., Barbu M.C., Petutschnigg A., Réh R., Krišťák Ľ. (2020). Analysis of larch-bark capacity for formaldehyde removal in wood adhesives. Int. J. Environ. Res. Public Health.

[19] Cateto C.A., Barreiro M.F., Rodrigues A.E. (2008). Monitoring of lignin-based polyurethane synthesis by FTIR-ATR. Ind. Crops Prod..

[20] Bajwa D.S., Pourhashem G., Ullah A.H., Bajwa S.G. (2019). A concise review of current lignin production, applications, products and their environment impact. Ind. Crops Prod..

[21] Mandlekar N., Cayla A., Rault F., Giraud S., Salaün F., Malucelli G., Guan J.-P. (2018). An Overview on the Use of Lignin and Its Derivatives in Fire Retardant Polymer Systems. Lignin—Trends and Applications.

[22] Zhang H., Bai Y., Yu B., Liu X., Chen F. (2017). A practicable process for lignin color reduction: Fractionation of lignin using methanol/water as a solvent. Green Chem..

[23] Evtuguin D.V., Andreolety J.P., Gandini A. (1998). Polyurethanes based on oxygen-organosolv lignin. Eur. Polym. J..

[24] Aristri M.A., Lubis M.A.R., Yadav S.M., Antov P., Papadopoulos A.N., Pizzi A., Fatriasari W., Ismayati M., Iswanto A.H. (2021). Recent Developments in Lignin- and Tannin-Based Non-Isocyanate Polyurethane Resins for Wood Adhesives—A Review. Appl. Sci..

[25] Thring R.W., Vanderlaan M.N., Griffin S.L. (1997). Polyurethanes from Alcell^®^ lignin. Biomass Bioenergy.

[26] Antov P., Savov V., Trichkov N., Krišťák Ľ., Réh R., Papadopoulos A.N., Taghiyari H.R., Pizzi A., Kunecová D., Pachikova M. (2021). Properties of high-density fiberboard bonded with urea–formaldehyde resin and ammonium lignosulfonate as a bio-based additive. Polymers.

[27] Alzagameem A., El Khaldi-Hansen B., Büchner D., Larkins M., Kamm B., Witzleben S., Schulze M. (2018). Lignocellulosic biomass as source for lignin-based environmentally benign antioxidants. Molecules.

[28] Réh R., Krišťák Ľ., Sedliačik J., Bekhta P., Božiková M., Kunecová D., Vozárová V., Tudor E.M., Antov P., Savov V. (2021). Utilization of birch bark as an eco-friendly filler in urea-formaldehyde adhesives for plywood manufacturing. Polymers.

[29] Bachtiar E.V., Kurkowiak K., Yan L., Kasal B., Kolb T. (2019). Thermal stability, fire performance, and mechanical properties of natural fibre fabric-reinforced polymer composites with different fire retardants. Polymers.

[30] Twite-Kabamba E., Mechraoui A., Rodrigue D. (2009). Rheological properties of polypropylene/hemp fiber composites. Polym. Compos..

[31] Rehman M., Gang D., Liu Q., Chen Y., Wang B., Peng D., Liu L. (2019). Ramie, a multipurpose crop: Potential applications, constraints and improvement strategies. Ind. Crops Prod..

[32] Mulyawan A.S., Sana A.W., Kaelani Z. (2015). Identifikasi Sifat Fisik Dan Sifat Termal Serat-Serat Selulosa Untuk Pembuatan Komposit. Arena Tekst..

[33] Yan L., Chouw N., Jayaraman K. (2014). Flax fibre and its composites—A review. Compos. Part B Eng..

[34] Yuan J.M., Feng Y.R., He L.P. (2016). Effect of thermal treatment on properties of ramie fibers. Polym. Degrad. Stab..

[35] Kalia S., Sheoran R. (2011). Modification of ramie fibers using microwaveassisted grafting and cellulase enzyme-assisted biopolishing: A comparative study of morphology, thermal stability, and crystallinity. Int. J. Polym. Anal. Charact..

[36] Liu X., Dai G. (2008). Impregnation of thermoplastic resin in jute fiber mat. Front. Chem. Eng. China.

[37] Xia C., Zhang S., Shi S.Q., Cai L., Huang J. (2016). Property enhancement of kenaf fiber reinforced composites by in situ aluminum hydroxide impregnation. Ind. Crop. Prod..

[38] Hermiati E., Risanto L., Lubis M.A.R., Laksana R.P.B., Dewi A.R. (2017). Chemical characterization of lignin from kraft pulping black liquor of Acacia mangium. AIP Conf. Proc..

[39] Ponnuchamy V., Gordobil O., Diaz R.H., Sandak A., Sandak J. (2021). Fractionation of lignin using organic solvents: A combined experimental and theoretical study. Int. J. Biol. Macromol..

[40] TAPPI (2007). T211 Ash in Wood, Pulp, Paper and Paperboard: Combustion at 525 °C.

[41] Templeton D., Ehrman T. (1995). Determination of Acid-Insoluble Lignin in Biomass—LAP-003.

[42] Ehrman T. (1996). Determination of Acid-Soluble Lignin in Biomass—LAP-004.

[43] Serrano L., Esakkimuthu E.S., Marlin N., Brochier-Salon M.C., Mortha G., Bertaud F. (2018). Fast, Easy, and Economical Quantification of Lignin Phenolic Hydroxyl Groups: Comparison with Classical Techniques. Energy Fuels.

[44] Aristri M.A., Lubis M.A.R., Laksana R.P.B., Fatriasari W., Ismayati M., Wulandari A.P., Ridho M.R. (2021). Bio-Polyurethane Resins Derived from Liquid Fractions of Lignin for the Modification of Ramie Fibers. J. Sylva Lestari.

[45] ASTM (2000). ASTM D 3379–75 Standard Test Method for Tensile Strength and Young’s Modulus for High-Modulus Single-Filament Materials.

[46] Sameni J., Krigstin S., Derval dos Santos R., Leao A., Sain M. (2014). Thermal characteristics of lignin residue from industrial processes. BioResources.

[47] Lubis M.A.R., Dewi A.R., Risanto L., Zaini L.H., Hermiati E. Isolation and Characterization of Lignin from Alkaline Pretreatment Black Liquor of Oil Palm Empty Fruit Bunch and Sugarcane Bagasse. Proceedings of the ASEAN COSAT 2014.

[48] Gordobil O., Herrera R., Poohphajai F., Sandak J., Sandak A. (2021). Impact of drying process on kraft lignin: Lignin-water interaction mechanism study by 2D NIR correlation spectroscopy. J. Mater. Res. Technol..

[49] Cardoso M., de Oliveira É.D., Passos M.L. (2009). Chemical composition and physical properties of black liquors and their effects on liquor recovery operation in Brazilian pulp mills. Fuel.

[50] Nikolskaya E., Janhunen P., Haapalainen M., Hiltunen Y. (2019). Solids Content of Black Liquor Measured by Online. Appl. Sci..

[51] Yotwadee H., Duangduen A., Viboon S. (2020). Lignin isolation from black liquor for wastewater quality improvement and bio-material recovery. Int. J. Environ. Sci. Dev..

[52] Jusuf P.G., Purwono S., Tawfiequrahman A. Permodelan Ekstraksi Lignin Mentah dari Black Liquor dengan Metode Asidifikasi pada pH Rendah. Proceedings of the Seminar Nasional Teknik Kimia “Kejuangan”.

[53] Sadeghifar H., Sadeghifar H., Ragauskas A., Ragauskas A., Ragauskas A., Ragauskas A. (2020). Perspective on Technical Lignin Fractionation. ACS Sustain. Chem. Eng..

[54] Sameni J., Krigstin S., Sain M. (2017). Solubility of Lignin and Acetylated Lignin in Organic Solvents. BioResources.

[55] Kubo S., Kadla J.F. (2005). Hydrogen bonding in lignin: A fourier transform infrared model compound study. Biomacromolecules.

[56] Li H., McDonald A.G. (2014). Fractionation and characterization of industrial lignins. Ind. Crops Prod..

[57] Buranov A.U., Ross K.A., Mazza G. (2010). Isolation and characterization of lignins extracted from flax shives using pressurized aqueous ethanol. Bioresour. Technol..

[58] Lucejko J.J., Tamburini D., Modugno F., Ribechini E., Colombini M.P. (2021). Analytical pyrolysis and mass spectrometry to characterise lignin in archaeological wood. Appl. Sci..

[59] Gogoi R., Alam M., Khandal R. (2014). Effect of increasing NCO/OH molar ratio on the physicomechanical and thermal properties of isocyanate terminated polyurethane prepolymer. Int. J. Basic Appl. Sci..

[60] Restasari A., Ardianingsih R., Abdillah L.H., Hartaya K. Effects of Toluene Diisocyanate’s Chemical Structure on Polyurethane’s Viscosity and Mechanical Properties for Propellant. Proceedings of the International Seminar on Aerospace Science and Technology.

[61] Gharib J., Pang S., Holland D. (2020). Synthesis and characterisation of polyurethane made from pyrolysis bio-oil of pine wood. Eur. Polym. J..

[62] Lubis M.A.R., Park B.D., Lee S.M. (2020). Microencapsulation of polymeric isocyanate for the modification of urea-formaldehyde resins. Int. J. Adhes. Adhes..

[63] Luo S., Gao L., Guo W. (2020). Effect of incorporation of lignin as bio-polyol on the performance of rigid lightweight wood–polyurethane composite foams. J. Wood Sci..

[64] Chen Y., Zhang H., Zhu Z., Fu S. (2020). High-Value Utilization of Hydroxymethylated Lignin in Polyurethane Adhesives.

[65] Amado J.C.Q. (2019). Thermal Resistance Properties of Polyurethanes and Its Composites. Thermosoftening Plastics.

[66] Wang Y.-Y., Wyman C.E., Cai C.M., Ragauskas A.J. (2019). Lignin-Based Polyurethanes from Unmodified Kraft Lignin Fractionated by Sequential Precipitation. ACS Appl. Polym. Mater..

[67] Tavares L., Stilhano C.R., Boas V. (2016). Bio-Based polyurethane prepared from Kraft lignin and modified castor oil. Express Polym. Lett..

[68] Trovati G., Sanches E.A., Neto S.C., Mascarenhas Y.P., Chierice G.O. (2009). Characterization of Polyurethane Resins by FTIR, TGA and XRD. J. Appl. Polym. Sci..

[69] Novarini E., Sukardan M.D. (2015). Potensi Serat Rami (Boehmeria Nivea S. Gaud) Sebagai Bahan Baku Industri Tekstil Dan Produk Tekstil Dan Tekstil Teknik. Arena Tekst..

[70] Bevitori A.B., da Silva I.L.A., Rohen L.A., Margem F.M., de Moraes Y.M., Monteiro S.N. Evaluation of Ramie Fibers Component by Infrared Spectroscopy. Proceedings of the 21st CBECIMAT—Congresso Brasileiro de Engenharia e Ciência dos Materiais.

[71] Simonassi N.T., Pereira A.C., Monteiro S.N., Muylaert F., De Deus J.F., Fontes C.M., Drelich J., Vermelha P., De Janeiro R., California P. (2017). Reinforcement of Polyester with Renewable Ramie Fibers. Mater. Res..

[72] Kandimalla R., Kalita S., Choudhury B., Devi D., Kalita D., Kalita K., Dash S., Kotoky J. (2016). Fiber from ramie plant (Boehmeria nivea): A novel suture biomaterial. Mater. Sci. Eng. C.

[73] Shahinur S., Hasan M., Ahsan Q., Haider J. (2020). Effect of Chemical Treatment on Thermal Properties of Jute Fiber Used in Polymer Composites. J. Compos. Sci..

[74] Shahinur S., Hasan M., Ahsan Q., Saha D.K., Islam M.S. (2015). Characterization on the Properties of Jute Fiber at Different Portions. Int. J. Polym. Sci..

[75] Tomczak F., Satyanarayana K.G., Sydenstricker T.H.D. (2007). Studies on lignocellulosic fibers of Brazil: Part III—Morphology fibers and properties of Brazilian curaua. Compos. Part. A.

[76] Bevitori A.B., Margem F.M., Carreiro R.S., Monteiro S.N., Calado V. Thermal Caracterization Behavior of Epoxy Composites Reinforced Ramie Fibers. Proceedings of the 67th ABM International Congress.

[77] Charlet K., Jernot J.P., Breard J., Gomina M. (2010). Scattering of morphological and mechanical properties of flax fibres. Ind. Crops Prod..

[78] Le Duigou A., Bourmaud A., Balnois E., Davies P., Baley C. (2012). Improving the interfacial properties between flax fibres and PLLA by a water fibre treatment and drying cycle. Ind. Crops Prod..

